# Supplementary material for: Splice-Junction-Based Mapping of Alternative Isoforms in the Human Proteome
Source: Cell Rep. Author manuscript; Available in PMC 2020 Jan 15. (PMC6961840; doi:10.1016/j.celrep.2019.11.026)
Supplement: 2 [file NIHMS1546469-supplement-2.zip › DF1/PXD010154/Lung/Lung_4_MYCBP2_SLLVPVDNIFNASQGVR.pdf]

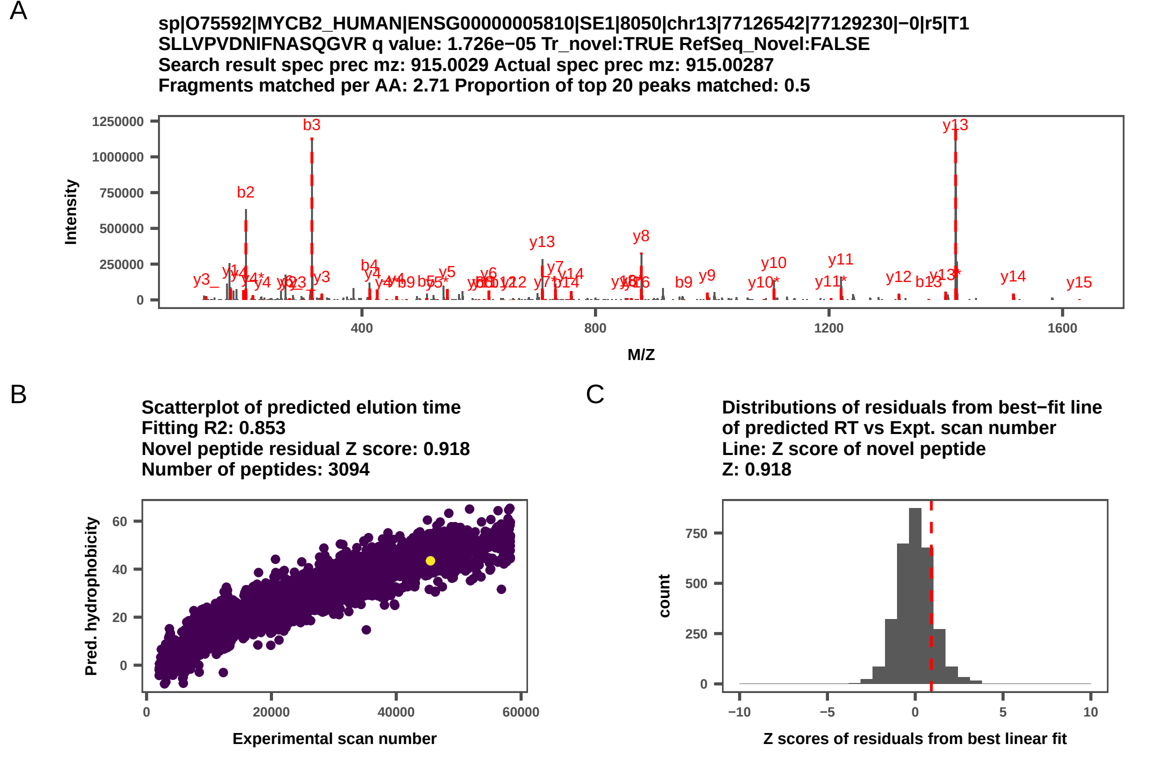
A
sp|O75592|MYCB2_HUMAN|ENSG00000005810|SE1|8050|chr13|77126542|77129230|−0|r5|T1
SLLVPVDNIFNASQGVR q value: 1.726e−05 Tr_novel:TRUE RefSeq_Novel:FALSE
Search result spec prec mz: 915.0029 Actual spec prec mz: 915.00287
Fragments matched per AA: 2.71 Proportion of top 20 peaks matched: 0.5
Intensity
M/Z
b2
b3
y13
y8
y10
y11
y12
y14
y15
b9
y9
B
Scatterplot of predicted elution time
Fitting R2: 0.853
Novel peptide residual Z score: 0.918
Number of peptides: 3094
Pred. hydrophobicity
Experimental scan number
C
Distributions of residuals from best−fit line
of predicted RT vs Expt. scan number
Line: Z score of novel peptide
Z: 0.918
count
Z scores of residuals from best linear fit
